# Investigating midwives and nurses reporting of ‘infant feeding at hospital discharge’: an online survey across NSW Australia

**DOI:** 10.1186/s13006-024-00637-w

**Published:** 2024-04-23

**Authors:** Lynne Henry, Elaine Burns, Rachel Jones, Lisa White, Michelle Simmons, Adrienne Kirby, Sarah J. Melov

**Affiliations:** 1 Women’s and Newborn Health, Westmead Hospital, Westmead, NSW Australia; 2 School of Nursing and Midwifery, Western Sydney University, Parramatta, NSW Australia; 3 Women’s Health Maternity, Blacktown and Mount Druitt Hospitals, Blacktown, NSW Australia; 4 NHMRC Clinical Trials Centre, The University of Sydney, Camperdown, NSW Australia; 5 Reproduction and Perinatal Centre, Faculty of Medicine and Health, The University of Sydney, Camperdown, NSW Australia; 6 Westmead Institute for Maternal and Fetal Medicine, Women’s and Newborn Health, Westmead Hospital, Westmead, NSW Australia

**Keywords:** Infant feeding, Breastfeeding, Monitoring, Electronic records, Hospital discharge, Health staff practice, Postpartum

## Abstract

**Background:**

The collection of data on ‘infant feeding at hospital discharge’ is used to monitor breastfeeding outcomes, health service benchmarking, and research. While some Australian states have clear definitions of this data collection point, there is no operational definition of ‘infant feeding at hospital discharge’ in the Australian state of New South Wales. Little is known about how midwives interpret the term ‘infant feeding at hospital discharge’, in particular, the timeframe used to calculate these important indicators. The purpose of this study was to explore midwives’ and nurses’ practices of reporting ‘infant feeding at hospital discharge’ in the Australian state of New South Wales.

**Methods:**

An online survey was distributed across public and private maternity hospitals in New South Wales, Australia. The survey asked midwives and nurses their practice of reporting ‘infant feeding at discharge’ from categories offered by the state Mothers and Babies report of either “*full breastfeeding”, “any breastfeeding”, and “infant formula only*”. The Qualtrics survey was available from December 2021 to May 2022.

**Results:**

There were 319 completed surveys for analysis and all 15 NSW Health Districts were represented. Some participants reported using the timeframe ‘*since birth’* as a reference (39%), however, the majority (54%, *n* = 173) referenced one of the feeding timeframes within the previous 24 h. Most midwives and nurses (83%, *n* = 265) recommended 24 h before discharge as the most relevant reference timeframe, and 65% (*n =* 207) were in favour of recording data on ‘exclusive breastfeeding’ since birth.

**Conclusion:**

This study identified multiple practice inconsistencies within New South Wales reporting of ‘infant feeding at hospital discharge’. This has ramifications for key health statistics, state reporting, and national benchmarking. While the Baby Friendly Hospital Initiative accreditation requires hospitals to demonstrate and continuously monitor at least a 75% exclusive breastfeeding rate on discharge, only 11 New South Wales facilities have achieved this accreditation. We recommend introducing an option to collect ‘exclusive breastfeeding’ on discharge’ which is in line with participant recommendations and the Baby Friendly Hospital accreditation. Other important considerations are the updated World Health Organization indicators such as, “*Ever breastfed”; “Early initiation of breastfeeding” (first hour); “Exclusively breastfed for the first two days after birth*”.

**Supplementary Information:**

The online version contains supplementary material available at 10.1186/s13006-024-00637-w.

## Background

Monitoring and collecting accurate breastfeeding rates, with defined indicators, are crucial for surveillance of population-level infant feeding practices, public health outcomes, and high-quality research [1–3]. Although the World Health Organization (WHO) and UNICEF recommends standardisation of breastfeeding indicators and infant feeding information [4, 5], nationally and internationally, data is collected and reported in different ways [6–9]. Variations even occur significantly between Australian states, for example, the Australian state of Victoria captures breastfeeding in hospital indicators such as ‘*any infant formal use in hospital’* to compare maternity services’ exclusive breastfeeding rates and report perinatal performance indicators [10]. The lack of agreed Australian standards of reporting infant feeding indicators not only limits policy makers and researchers, it poses a significant challenge when comparing international outcomes [9, 11].

### ‘Infant feeding at hospital discharge’ New South Wales Australia

Both midwives and nurses in the state of New South Wales (NSW), Australia, record data on ‘infant feeding at hospital discharge’ in hospital electronic databases. The state-wide ‘Perinatal Data Collection’ (PDC) is populated from this data and informs the annual NSW ‘Mothers and Babies’ report [12]. Within the NSW maternity database, the categories for ‘infant feeding at hospital discharge’, are *‘full breastfeeding’*, *‘any breastfeeding’*, and *‘infant formula only’*. However, the associated data dictionary offers no definition of these indicators or timeframe to reference.

Data from the NSW Mothers and Babies report for 2020 reveals a consistent decline in ‘full breastfeeding’ in NSW since 2016, from 82.6% down to 74.1% [12]. However, it is unknown if there is consensus amongst NSW midwives and nurses who enter this data, for example, whether ‘*full breastfeeding’* at hospital discharge reflects breastfeeding since birth, or only breastfeeding on the day of discharge, or refers to the last feed prior to discharge. Due to the absence of guidance, the reported NSW ‘infant feeding at hospital discharge’ data may be inconsistently reported and open to varied interpretations of the timeframe to reference.

There are many accounts nationally and internationally of inadequacies in general breastfeeding data collected, and the interpretation of this data [7, 13]. To date, we are unaware of any contemporary literature that examines midwives’ and nurses’ practice regarding timeframe interpretation of current indicators when reporting ‘infant feeding at hospital discharge’. The aim of this study was to explore how NSW midwives and nurses interpret and report ‘infant feeding at hospital discharge’ when entering data into the relevant database. A secondary aim was to ask midwives and nurses to recommend a relevant operational definition of ‘infant feeding at hospital discharge’.

## Methods

### Study design

An online survey was developed based on the questions asked about ‘infant feeding at hospital discharge’ for the state-wide PDC statistics. The anonymous online survey was distributed through established professional networks, and social media, to midwives and nurses who met the inclusion criteria. The inclusion criteria for this study were participants over 18 years of age, and currently working as a midwife or nurse with responsibility for completing hospital maternity discharge data in NSW.

### Survey development and questions

Survey questions were developed based on the categories reported in the NSW Mothers and Babies report of “*full breastfeeding”, “any breastfeeding”, and “infant formula only*” and the potential timeframes were developed from a hospital-based quality improvement audit (reviewing midwifery discharge practices). This ward-based quality audit was conducted in an NSW postnatal unit over one month (June 2021) and was designed to assess local midwifery level understanding of what ‘infant feeding at hospital discharge’ meant to them. Midwives were recruited for the quality audit survey with a local flyer and through snowball sampling. We identified inconsistencies in practice through this audit and, therefore sought to gain a broader understanding of infant feeding at discharge data collection practices. The quality audit findings not only prompted further investigation but also provided a strong foundation for survey development.

In total, twenty-six survey questions were developed; fifteen were closed-ended, which gathered demographic characteristics along with breastfeeding education and experience. There were seven multiple choice questions based on potential timeframes, and a further three questions based on a five-point Likert scale response from strongly agree to strongly disagree, with one final open text question. The survey was pilot tested over two weeks, by a convenience sample of ten midwives who volunteered to contribute to the survey development following involvement in the quality audit. The pilot group of ten midwives were a mixture of five senior midwives, with ten or more years of experience, three had five years or less experience, and two were newly graduated within the past two years. All midwives who contributed to the pilot survey regularly discharged mothers and babies from hospital. Small changes were made to the survey following the collation of evaluation comments from the pilot group. These included recommendations to refine some wording of questions to improve readability. The pilot testing also confirmed the mean time to complete the survey (< 10 min). The definitive version received ethics approval before being uploaded to the secure online Qualtrics survey platform. The Qualtrics survey was available from December 2021 to May 2022. The survey is available as an additional file. (Additional file 1.)

The online survey gathered general participant characteristics, qualifications, and years of experience completing discharge documentation for women leaving hospital after giving birth. Additionally, survey questions asked participants to share their current discharge reporting practice and interpretation of the infant feeding categories used in the PDC: *‘full breastfeeding’, ‘any breastfeeding’*, and ‘*infant formula only’* [12]. In order to meet the study aim, survey participants were asked what reference timeframe they would recommend as ‘clinically relevant’ at discharge. Participants were also asked if the introduction of the WHO definition ‘*exclusive breastfeeding’* would be a relevant category option for routine data collection at discharge.

### Setting and recruitment

At the time of writing, the state of NSW had the largest population in Australia, with approximately 8.341 million people accounted for in 2023 [14]. The majority of NSW women birth in public hospitals, (79% *n =* 72,116) with approximately 91,240 births per year [15]. The state of NSW is divided administratively into 15 local health districts inclusive of both metropolitan and rural districts. The majority of NSW hospitals utilise the eMaternity electronic data system (58%) [15]. Cerner, Meditech and other systems supply the remainder of PDC records [15].

Midwives and nurses who discharge postnatal women and infants from maternity facilities in NSW were the targeted population for recruitment. All NSW local health districts, including both public and private hospitals, were invited to participate. A flyer with a QR code and link to the Qualtrics survey was developed to advertise the study. The QR code, or survey link, opened the landing page, where information on the research was offered. Clicking on the ‘agree to participate’ button confirmed consent. Snowball sampling was used to distribute the survey using professional networks and social media platforms. Information on the study was also disseminated at the NSW State Clinical Midwifery Consultant group meetings with approximately 120 state-wide contacts. Whilst auditing the data, there were no duplicate IP addresses detected. Given the ongoing demands of clinical work in acute hospital environments, it is unlikely participants would repeatedly complete the survey.

### Statistical analysis

Survey data was cleaned and sorted using Excel and entered *SAS 9.4* (SAS Institute Inc., Cary, NC, USA) for analysis. Survey response variables were summarised as mean and standard deviation, if normally distributed, and median and interquartile range if not. Categorical variables are presented as frequency (%) in relevant categories. Summary statistics are presented as total respondents and grouped into districts and individual hospitals. Individual hospitals with less than 10 responses were grouped together as “other”. Midwives with additional education and qualifications related to breastfeeding, who had worked in breastfeeding support for more than 5 years, were grouped together in a ‘lactation specialist’ category. This grouping occurred prior to analysis. Groups were compared with a Wilcoxon rank sum test [16]. Binary variables were summarised with percentages and compared with a Pearson χ2, or Fisher’s exact test, as appropriate. Inductive content analysis was used to detect patterns in the final open-text response question, in order to gain a deeper understanding of participants’ views [17].

## Results

A total of 360 people accessed the survey, with most using the online anonymous link (*n* = 245) and others using the flyer with QR code (*n* = 115). Three people did not consent after reading the participant information, and surveys with less than 90% completion were excluded (*n* = 38). In total 319 completed surveys were available for analysis. Both midwives and nurses from 48 public and 16 private hospitals in NSW participated. Participants represented all 15 NSW local health districts. A similar representation was noted from Sydney metropolitan (51% *n =* 164) and NSW regional (49% *n =* 155) areas. There were 8% (*n =* 27) of participants who met the study protocol criteria to be considered a ‘lactation specialist’.

### Participant characteristics and discharge practice

Of the 319 included participants, most were midwives (84% *n* = 267), and the remainder were nurses or students (16% *n* = 52). Most midwives were working permanently on the postnatal ward or working in rotational roles throughout maternity (52% *n* = 167). A high number of participants (95% *n =* 302) were in a position that both discharged women and provided breastfeeding support. More than one-third of participants said they had provided breastfeeding support for ten years or more (44% *n =* 140). The majority of participants received their professional qualification in Australia (93% *n =* 297) and held a bachelor’s (53% *n =* 168) or master’s degree (15% *n =* 47).

A comparison between Sydney metropolitan and regional areas revealed similar characteristics except that more of the metropolitan cohort were aged 40 years or younger (50%) compared to regional responders (33%) (Table 1).

**Table 1:** Participant characteristics by metropolitan and regional areas in the state of New South Wales, Australia

Characteristic		Regional	Sydney Metro	
		*n* (%)	*n* (%)	Total
Age (*n* = 319)	< 30 yrs.	19 (12)	25 (15)	44
	30–40 yrs.	32 (21)	57 (35)	89
	41–50 yrs.	43 (28)	34 (21)	77
	> 50 yrs.	61 (39)	48 (29)	109
First professional	Australia	148 (95)	149 (91)	297
Registration (*n* = 297)	United Kingdom	3 (2)	7 (4)	10
	Other	4 (3)	8 (5)	12
Highest qualification	Current student	2 (1)	5 (3)	7
(*n* = 168)	Certificate	9 (6)	21 (13)	30
	Grad Dip/Certificate	16 (10)	12 (7)	28
	Diploma	19 (12)	17 (10)	36
	Bachelor	80 (52)	88 (54)	168
	Masters	27 (17)	20 (12)	47
	Other	1 (1)		1
	PhD	1 (1)	1 (1)	2
Main area of work	Lactation Consultant		2 (1)	2
	Continuity of care model	18 (12)	8 (5)	26
	Antenatal Clinic	7 (5)	3 (2)	10
	Birth unit	10 (6)	15 (9)	25
	Postnatal ward	24 (15)	46 (28)	70
	NICU/SCN	9 (6)	18 (11)	27
	Rotational midwife	61 (39)	36 (22)	97
	Midwifery in the home	4 (3)	13 (8)	17
	Student midwife/nurse	2 (1)	4 (2)	6
	Other	20 (13)	19 (12)	39
Clinical role	Clinical midwifery consultant	14 (9)	14 (9)	28
	Clinical midwifery specialist	4 (3)	1 (1)	5
	Midwife educator	12 (8)	7 (4)	19
	Midwife	100 (65)	104 (63)	204
	Manager	6 (4)	5 (3)	11
	Nurse	4 (3)	14 (9)	18
	Nurse educator	2 (1)	4 (2)	6
	Other	11 (7)	8 (5)	19
	Student nurse/midwife	2 (1)	7 (4)	9
Years practiced	< 5	63 (41)	71 (43)	134
	5–9	30 (19)	38 (23)	68
	≥ 10	62 (40)	55 (34)	117
Provides breastfeeding support (*n* = 319)	No	9 (6)	8 (5)	17
	Yes	146 (94)	156 (95)	302
Years providing breastfeeding support	< 5	41 (28)	54 (35)	95
	5–9	29 (20)	38 (24)	67
	≥ 10	76 (52)	64 (41)	140
IBCLC	No	118 (76)	120 (73)	238
	Previously but not re-certified	12 (8)	6 (4)	18
	Working towards	6 (4)	14 (9)	20
	Yes- currently certified	19 (12)	24 (15)	43

NICU/SCN = Neonatal Intensive Care/ Special Care Nursery IBCLC = International Board-Certified Lactation Consultant

A high number of participants (66% *n =* 210) stated they had some clinical contact with the postnatal women they were discharging either ‘some’, or ‘most’, of the time. However, 29% (*n =* 91) of participants disclosed, that ‘some’ or ‘most’ of the time they had no clinical contact with the mother or infant before completing the computer-based discharge data. Midwives and nurses who had no contact with the mother, 67% (*n =* 61) reported collecting discharge feeding information from the clinical notes. Most participants 70% (*n =* 223) reported that they felt confident ‘most of the time’ with the accuracy of the information they were reporting. Therefore approximately 30% of participants disclosed less confidence in the accuracy of the information they provided reporting ‘infant feeding at hospital discharge’.

### Reference timeframe currently used by participants to define ‘infant feeding at hospital discharge’

Participants were asked to select one of five timeframe reference options to describe their current infant feeding discharge practice. Options available included: ‘*feeding over the previous 24 h’*, ‘*feeding over the previous 12 h’*, ‘*most recent two or three feeding episodes’*, ‘*most recent single feeding episode’*, and ‘*all feeding since birth’*. There were ‘other’ and free text responses also available. Across all local health districts and hospitals, no single reference timeframe was used by all midwives and nurses. In total, 54% (*n =* 173) of participants currently reported feeding at discharge by using a reference timeframe within the last 24 h (Table 2). This is an amalgamation of the 33% (*n* = 105) who ticked ‘*feeding over the previous 24 h*’ timeframe, and those who ticked the shorter timeframe of ‘*feeding over the previous 12 hours*’ (8% *n =* 27), or ‘*most recent two or three feeds’* (11% *n* = 35) and ‘*most recent single feeding episode’* (2% *n* = 6). Other responses revealed that 39% (*n =* 126) of midwives and nurses currently use a reference timeframe of ‘*all feeding from birth’* when entering discharge infant feeding information data into medical records (Table 2).

**Table 2:** Timeframe currently used by participants by Local Health Districts, New South Wales, Australia

Local Health District	Total	Most recent single feeding episode	Most recent two or three feeding episodes	Feeding over the previous 12 h	Feeding over the previous 24 h	All feeding since birth	Other
		*n* (%)	*n* (%)	*n* (%)	*n* (%)	*n* (%)	*n* (%)
Sydney	8			1 (13)	4 (50)	3 (38)	
South-Eastern Sydney	26		3 (12)	3 (12)	7 (27)	10 (38)	3 (12)
South-Western Sydney	25		1 (4)	1 (4)	10 (40)	12 (48)	1 (4)
Western Sydney	73	1 (1)	12 (16)	5 (7)	25 (34)	26 (36)	4 (5)
Northern Sydney	16	1 (6)	1 (6)	4 (25)	2 (13)	7 (44)	1 (6)
Nepean Blue Mountains	16	1 (6)	2 (13)		7 (44)	5 (31)	1 (6)
Illawarra Shoalhaven	32	1 (3)	5 (16)	1 (3)	12 (38)	11 (34)	2 (6)
Murrumbidgee	26			1 (4)	10 (38)	12 (46)	3 (12)
Central Coast	22		3 (14)	4 (18)	6 (27)	9 (41)	
Hunter New England	25	1 (4)	4 (16)	1 (4)	10 (40)	8 (32)	1 (4)
Mid North Coast	3					3 (100)	
Northern NSW	10		2 (20)	1 (10)	4 (40)	3 (30)	
Southern NSW	16		1 (6)	2 (13)	5 (31)	6 (38)	2 (13)
Western NSW	18	1 (6)		2 (11)	3 (17)	10 (56)	2 (11)
Far West	3		1 (33)	1 (33)		1 (33)	
Total	319	6 (2)	35 (11)	27 (8)	105 (33)	126 (39)	20 (6)

The free text option was used by 6% (*n =* 20) of participants to describe the timeframe they currently used to define feeding at discharge. A variety of responses were provided that included: *“what the mother intends to do”*, *“feeding plan the baby is being discharged on*”, and *“the previous 2–3 days”.* There was no difference in current reference timeframe practices between lactation specialists and non-specialists (*p* = 0.14) or metropolitan or regional districts (*p* = 0.99).

### Participants recommendations for a reference timeframe to define ‘infant feeding at hospital discharge’

The majority of participants (59% *n =* 187) recommended a clinically relevant reference timeframe for ‘infant feeding at hospital discharge’ as ≤ 24 h prior to hospital discharge. When combined with those who recommended a reference timeframe of; ‘*most recent feed’ n =* 9 (3%), *‘most recent 2–3 feed’ n =* 34 (11%), *and ‘≤12 hours’ n =* 35 (11%), this brought the total to 83% (*n =* 265) who identified a preferred reference timeframe that was *within* 24 hours. There were 16% (*n =* 50) of participants who stated the most relevant reference timeframe for feeding at discharge is ‘*all feeding since birth’* (Tables 3 & 4).

**Table 3:** Timeframe recommended by participants by Local Health Districts New South Wales, Australia

Local Health District	Total	Most recent single feeding episode	Most recent two or three feeding episodes	Feeding over the previous 12 h	Feeding over the previous 24 h	All feeding since birth
	*n*	*n* (%)	*n* (%)	*n* (%)	*n* (%)	*n* (%)
Sydney	8				7 (88)	1 (13)
South-Eastern Sydney	26	1 (4)	3 (12)	4 (15)	14 (54)	4 (15)
South-Western Sydney	24		3 (12)		16 (64)	5 (20)
Western Sydney	73	1 (1)	11 (15)	6 (8)	45 (62)	10 (14)
Northern Sydney	15	1 (6)	1 (6)	3 (19)	8 (50)	2 (13)
Nepean Blue Mountains	15	2 (13)	1 (6)	3 (19)	6 (38)	3 (19)
Illawarra Shoalhaven	31	2 (6)	2 (6)	1 (3)	20 (63)	6 (19)
Murrumbidgee	26			3 (12)	17 (65)	6 (23)
Central Coast	22		4 (18)	6 (27)	8 (36)	4 (18)
Hunter New England	25	1 (4)	4 (16)	2 (8)	13 (52)	5 (20)
Mid North Coast	3	1 (33)			2 (67)	
Northern NSW	10		2 (20)	1 (10)	6 (60)	1 (10)
Southern NSW	16		1 (6)	4 (25)	10 (63)	1 (6)
Western NSW	18		1 (6)	2 (11)	13 (72)	2 (11)
Far West	3		1 (33)		2 (67)	
Total	315	9 (3)	34 (11)	35 (11)	187 (59)	50 (16)

**Table 4:** Reference timeframe currently used, and timeframe recommended by midwives and nurses as an operational definition

Timeframe reference	Current reference *n* (%) Total = 319	Recommended reference *n* (%) Total = 315
≤ 24 h grouping		
Most recent feed	6 (2)	9 (3)
Most recent 2–3 feed	35 (11)	34 (11)
≤ 12 h	27 (8)	35 (11)
≤ 24 h	105 (33)	187 (59)
All feeds since birth	126 (39)	50 (16)
Other	20 (6)	-

The findings revealed some consistency between participants’ recommendations and current practice for *‘< 12 hours’ timeframe’, ‘recent 2–3 feeds’ and ‘recent single feed’* reference timeframes (Table 4). However, there was marked inconsistency between the groups who currently use ‘*all feeding since birth’* timeframe (39% *n* = 126) and those who would recommend this timeframe (16% *n =* 50). Similarly, while most participants recommended the ‘*previous 24 hours’* timeframe (59% *n =* 187) for an accurate measure of feeding at discharge, only 33% (*n =* = 105) used this timeframe in practice (Table 4).

### Reference timeframe currently used by participants to define ‘full breastfeeding’ at hospital discharge

Participants were asked to choose from five options of what ‘*full breastfeeding’* at discharge means. Three responses were equally weighted, that is; 29% (*n =* 91) of participants identified ‘*full breastfeeding’* as ‘*only breastfeeding or breastmilk feeding since birth’*, while 29% (*n* = 93) said ‘*only breastfeeding or breastmilk feeding over the previous 24 hours’* and, 28% (*n* = 90) recorded ‘full breastfeeding’ as ‘*only breastfeeding on the day of discharge’* (Table 5). Other responses for what ‘full breastfeeding at discharge’ meant included: “*mothers’ intention to feed her infant when at home”* (3% *n =* 10), and 7% (*n =* 23) stated ‘*only breastfeeding or breastmilk feeding over the previous 12 hours*’. Across all NSW Local Health Districts, there were inconsistencies in what *‘full breastfeeding at discharge’* meant (Table 5).

**Table 5:** Timeframe used for ‘full breastfeeding at hospital discharge’ by Local Health Districts New South Wales

Local Health District	Total	Mothers’ intention at discharge	On the day of discharge	Over the last 12 h	Over the previous 24 h	Since birth	Question not answered
		*n* (%)	*n* (%)	*n* (%)	*n* (%)	*n* (%)	*n* (%)
Sydney	8		1 (13)		5 (63)	1 (13)	1 (13)
South-Eastern Sydney	26	1 (4)	8 (31)	3 (12)	4 (15)	8 (31)	2 (7)
South-Western Sydney	25		9 (36)	1 (4)	5 (20)	8 (32)	2 (8)
Western Sydney	73	6 (8)	17 (23)	8 (11)	21 (29)	20 (27)	1 (2)
Northern Sydney	16		5 (31)	1 (6)	5 (31)	5 (31)	
Nepean Blue Mountains	16		4 (25)	1 (6)	4 (25)	4 (25)	3 (19)
Illawarra Shoalhaven	32	2 (6)	7 (22)	2 (6)	10 (31)	8 (25)	3 (10)
Murrumbidgee	26		3 (12)	1 (4)	11 (42)	11 (42)	
Central Coast	22	1 (5)	9 (41)	1 (5)	5 (23)	6 (27)	
Hunter New England	25		5 (20)	3 (12)	10 (40)	7 (28)	
Mid North Coast	3				2 (67)	1 (33)	
Northern NSW	10		3 (30)	1 (10)	3 (30)	3 (30)	
Southern NSW	16		10 (63)		3 (19)	3 (19)	
Western NSW	18		8 (44)	1 (6)	4 (22)	5 (28)	
Far West	3		1 (33)		1 (33)	1 (33)	
TOTAL	319	10 (3)	90 (28)	23 (7)	93 (29)	91 (29)	12 (4)

### Exclusive breastfeeding as a category definition to report

Participants were asked if the WHO-defined term ‘*exclusive breastfeeding’* was relevant data to collect at discharge. Overall, 65% (*n =* 207) of participants recommended *‘exclusive breastfeeding’* as a category definition to reference at discharge. In total, 65% of participants either *strongly ‘agreed’* (39% *n =* 121), or ‘*agreed’* (27% *n* = 86) with the statement.

### Free text additional comments on ‘infant feeding at hospital discharge’

In the final survey question ‘Do you have any further comments or suggestions on ‘feeding at discharge?’ a total of 72 participants left a short response. Content analysis of the one to two-sentence responses revealed participant concerns about lack of resources, lack of clear definitions, and the lack of data collection on ‘feeding intention’ after discharge.

Concerns included the lack of dedicated lactation consultants, on postnatal wards, to support women with more complex breastfeeding challenges. Discharging women without having met the mother, and baby, was raised as inappropriate, but necessary, practice in the busy postnatal ward environment. Some of the responses endorsed previous answers to closed-ended survey questions. For example, participants added comments such as the importance of clear and simple definitions and checking with the woman:

*“Clear definition is essential, so everyone is doing/measuring the same thing” (P47)*.

*“I think specifying a time frame would be great - I think 24hrs is most relevant as generally babies from NICU may have had formula but…labelling as mixed feeding is not appropriate if they have transitioned back to breast milk by discharge.” (P21)*.

*“Make it as basic as possible. Feeding at discharge is literally that. If in doubt, ask the mother!” (P32)*.

Some participants highlighted the need for definitions for measuring rates against other jurisdictions:

*“Being clear about ‘any’ or ‘exclusive’ is important for BFHI* [Baby Friendly Hospital Initiative] *data collection” (P167).*

The most common additional open text response was the suggestion that breastfeeding at discharge should include an additional option for collecting data on the mother’s intention for infant feeding after discharge from hospital:

*“There should definitely be obvious delineation however - for example, if a baby is BF* [breastfeeding]*with formula top-ups to maintain BGLs* [blood glucose levels] *and then exclusively breastfed from there on. If they are discharged later that same day, I would normally put BF* [breastfeeding] *on discharge because that is their plan. I think I go more with the intention, i.e., ‘What is the plan for this baby?‘” (P110)*.

*“Perhaps a section about feeding intentions at discharge. Some parents are only BF*, [breastfeeding] *but then go home and commence formula top-ups on their own” (P24)*.

*“Feeding over 24 h before discharge and feeding intention of the parents after discharge” (P105)*.

## Discussion

Midwives and nurses in NSW who discharge postnatal women from hospitals are required to categorise ‘infant feeding at hospital discharge’ but are not provided with definitions of indicators or timeframe to reference. It is well understood that infant feeding indicators are important measures to track progress and guide health service delivery [18, 19], however the results of our study show there was no NSW hospital-level, or region-level, consistency when midwives and nurses reported ‘infant feeding at hospital discharge’. This raises concerns about the accuracy of the data recorded when discharging women and infants from NSW hospitals.

While neighbouring state, Victoria provides consistent data on breastfeeding hospital indicators such as “*rate of use of infant formula in hospital by breastfed babies born at ≥ 37 weeks’ gestation”* [10], there is no national standard for state comparisons. As a result, unreliable data from NSW not only impacts NSW key state health statistics, but national outcomes.

Nearly two decades ago, a descriptive study examined newborn feeding practices at the time of discharge from NSW hospitals [20]. This study collected data from the 2007 NSW midwives’ data collection systems used at the time, which similarly classified infant feeding at hospital discharge as ‘*full breastfeeding’ ‘partial breastfeeding’*, and *‘not breastfeeding’*. Even then, limitations acknowledged in this paper was the questionable validity or accuracy of reporting infant feeding practice at hospital discharge [20].

The framework developed by the WHO and UNICEF called for international standards of infant feeding data collection policy, and already over seventy countries gather specific infant feeding practice data [4, 11]. This was highlighted as a priority for the 2019 Australian National Breastfeeding Strategy (ANBS), but we would like to point out that no action has been taken to achieve the ANBS goals. Although steps were taken to establish a national breastfeeding advisory committee [21] to date, no official meetings have been held [11].

### Barriers reported when collecting ‘Infant feeding at hospital discharge’ data

Identifying and resolving clinical and organisational barriers that hinder the collection of accurate data is complex [22]. Poor staffing, heavy workloads, and limited protected time to complete discharge data were reported by midwives and nurses as barriers to accurate data collection. Busy postnatal wards rely on midwives and nurses to discharge women and their infants in electronic notification systems after limited contact with the women they discharge, or in some cases no contact. Discharge information is gathered from patient notes and clinical handovers in sometimes pressured working environments. This could impact the accuracy of the data, therefore one strategy offered by participants in this study was simply “If in doubt ask the mother”. The results of this research support this approach and we argue that ‘infant feeding at hospital discharge' data should be collected at the bedside, in consultation with the mother, as this will enable the most accurate account of infant feeding practice.

### Midwives and nurses’ confidence level reporting discharge data

Midwives and nurses who responded to this survey revealed inconsistent reporting of ‘infant feeding at hospital discharge’ data with many variations in practice. Some participants disclosed low confident in the accuracy of the information they provided on ‘infant feeding at hospital discharge’. Implications for practice were further evident when one-third of midwives and nurses identified some or most of the time, they had no clinical contact with the mother or infant they were entering data for. These findings are of concern because health care relies on midwives and nurses feeling confident with the information they are documenting [22, 23].

### The recommended timeframe for recording infant feeding at discharge

The findings from this study revealed a variety of reference timeframes used to describe ‘infant feeding at hospital discharge’ including all feeding since birth, the last 12 h, and the last few feeds before discharge. However, most midwives and nurses who participated in this study (83%) recommended a clinically relevant operational definition to be infant feeding within the last 24 h before discharge.

While using the last 24 h to gather information is in line with the 2021 updated WHO indicators for collecting data from women, this is related to the most accurate recall time for mothers [4]. The WHO recommend other measurements such as, “*Ever breastfed”; “Early initiation of breastfeeding” (first hour); “Exclusively breastfed for the first two days after birth*” [4] which we argue are more relevant for a hospital setting.

There were participants who suggested ‘infant feeding at hospital discharge’ should include the mothers’ intentions of feeding on discharge. While mothers’ intention of feeding is an important antenatal indicator, birth and early postnatal events can alter mothers original intentions, therefore a not a reliable ‘infant feeding at hospital discharge’ indicator [24].

The other area of inconsistency identified in this study is the lack of a clear understanding of ‘full breastfeeding’. Results from this research show 65% of participants recommended introducing an option to report ‘exclusive breastfeeding’ at discharge.

### Clarifying the differences between ‘full breastfeeding’ and ‘exclusive breastfeeding’

Collecting data on ‘full breastfeeding’ revealed that some midwives and nurses recorded this as only breastfeeding since birth, or only breastfeeding on the day of discharge, or what the mother intends to feed when she returns home. These varied interpretations mean that the indicator results do not accurately reflect ‘full breastfeeding’ because infants who have been mixed fed could be represented in the ‘full breastfeeding’ statistics. Labbok argued in the 1990s that ‘full breastfeeding’ should be broken into categories that describe ‘exclusive breastfeeding’ and ‘almost exclusive’ [25]. This older research highlighted confusion around the term ‘full breastfeeding’ even then, which potentially derailed accurate data collection [25]. In comparison, an option to record ‘exclusive breastfeeding’ since birth, or even exclusive breastfeeding in the first two days after birth, as recommended by WHO [4], has the potential to improve hospital data collection around infant feeding practices. In our study, 65% of participants recommended ‘*exclusive breastfeeding’* as an important indicator to record at discharge.

### The case for Baby-Friendly Hospital Initiative

The Baby Friendly Hospital Initiative (BFHI), accreditation requires facilities to demonstrate and continuously monitor at least a 75% exclusive breastfeeding rate on discharge [26]. This means gaining BFHI accreditation not only promotes, protects, and supports breastfeeding as the normal way to feed infants, it also supports health facilities, midwives, and nurses to improve the consistency and accuracy of data collection [27]. At the time of writing only 11 facilities within NSW were BFHI accredited. The original audit, that informed our study, was performed at a facility that was not BFHI accredited. We argue that clear definitions with timeframes in line with WHO and BFHI standards should be added to all NSW database dictionaries for midwives and nurses to use while discharging mothers and their infants from NSW hospitals.

### Strengths of the study

One strength of this study was identifying the inconsistency in practice through a workplace audit and developing an exploratory study to gain a deeper understanding of current practice as a result. We considered the importance of equity of access for regional areas and smaller facilitates interested in participating in this research and therefore the use of an online survey was considered a strength. Data were provided from all health districts throughout the state of NSW with equal representation of regional and metropolitan participants.

### Limitations of the study

Data for this study were collected from one Australian state, NSW, and may not represent the discharge data collected in other states and territories. There was no validated survey tool available which is also a limitation of the study. Additionally, participants self-selected to complete the survey introducing potential sampling bias, as participants may have been the professionals most interested in the subject. Whilst the aim of this study was to interpret what ‘feeding at discharge’ meant to midwives and nurses, irrespective of the amount of time women stay in hospital after birth, we acknowledge the length of postnatal stay may alter participants’ interpretations. Although highlighted as a limitation of this study, this important point is likely to promote further investigation.

## Conclusion

This study has highlighted inconsistencies when NSW midwives and nurses report infant feeding at hospital discharge. Without clearly defined indicators there are distortions of breastfeeding data, which has ramifications for state reporting and national benchmarking. Protecting and promoting breastfeeding is a public health issue which depends on accurate data to inform standards and targeted intervention programs. We advocate for the introduction of an operational definition of ‘infant feeding at hospital discharge’ that goes beyond ‘full breastfeeding’ to incorporate ‘exclusive breastfeeding’ reflecting WHO indicators and BFHI recommendations. We recommend that defined indicators be implemented into all NSW databases for midwives and nurses to use. Further recommendations on improving practice are to introduce as a standard of care for midwives and nurses to gather ‘infant feeding at hospital discharge’ data at the mother’s bedside.

Replicating this research on a wider national and even international level will offer health agencies an understanding of how ‘infant feeding at discharge’ indicators vary between state, national, and international data custodians. Result of this study highlight an urgent need for Australia to establish national standards of ‘infant feeding at hospital discharge’ indicators, thereby improving the accuracy of this important breastfeeding indicator.

### Electronic supplementary material

Below is the link to the electronic supplementary material.

Supplementary Material 1

## Data Availability

Not applicable.
