# Supplementary material for: Investigating midwives and nurses reporting of ‘infant feeding at hospital discharge’: an online survey across NSW Australia
Source: Int Breastfeed J. 2024 Apr 23;19:29. doi: 10.1186/s13006-024-00637-w (PMC11040791; doi:10.1186/s13006-024-00637-w)
Supplement: Supplementary file 1 — Supplementary Material 1 [file 13006_2024_637_MOESM1_ESM.pdf]

Questionnaire

Investigating midwives and nurses reporting of 'infant feeding at hospital discharge': an online survey across NSW Australia

<u>Section A</u>

Demographics

The following questions are about you and what you do. There are no right or wrong answers.

What Local Health District do you currently practice in?

Sydney
South-Western Sydney
South-Eastern Sydney
Illawarra Shoalhaven
Western Sydney
Nepean Blue Mountains
Northern Sydney
Central Coast
Hunter New England
Northern NSW
Mid North Coast
Southern NSW
Murrumbidgee
Western NSW
Far West
Other

What hospital within this district do you work?

Public
Armidale
Auburn
Bankstown-Lidcombe
Bathurst Base
Bega Base
Blacktown
Blue Mountains
Bowral
Broken Hill Base
Campbelltown
Canterbury
Coffs Harbour
Dubbo Base
Fairfield
Gosford
Grafton Base
Griffith Base
Hawkesbury
Hornsby
Inverell
John Hunter
Kempsey

Lismore Base
Liverpool
Maitland
Manning Base
Moruya
Mudgee
Nepean
Northern Beaches
Orange Base
Port Macquarie Base
Queanbeyan
Royal Hospital for Women
Royal North Shore
Royal Prince Alfred
Shoalhaven
St. George
Sutherland
Tamworth Base
Tweed Heads
Westmead
Wollongong
Other public hospital

Private
Calvary
Hurstville Community
Karena Private
Mater,
Nepean Private
Newcastle Private
North Gosford Private
North Shore Private
North Sydney
Norwest Private
Prince of Wales Private
St. George Private
Sydney Adventist
Sydney Southwest Private
Wagga Wagga
Westmead Private
Wollongong Private
Other private hospital:

How old are you?

<30 yrs.
30 – 40 yrs.
41-50 yrs.
>50 yrs.

What is your highest clinical qualification?

Certificate
Diploma
Bachelor
Post Grad
Current student midwife/nurse
Other

How many years have you been registered as a midwife/nurse?

Free text

What country were you first registered in?

United Kingdom
New Zealand
Philippines
India
Other Free text

What is your main area of work?

Continuity of care model
Rotational midwife
Birth unit
Antenatal Clinic
Postnatal ward
Midwifery in the home
NICU/SCN/ NCN
Student midwife/nurse
Other

What is your current clinical role?

Student nurse/midwife
Midwife
Nurse
Midwife educator
Nurse educator
Clinical Midwife Consultant
Clinical Nurse Consultant
Manager
Other

How many years have you practiced in this role?

< 5yrs
≥ 5yrs
≥ 10yrs

Contracted hours at your facility?

Part-time
Full-time
Casual
Other

Section B
Breastfeeding

This section is about your experience and education related to breastfeeding.

Do you have a role that provides breastfeeding support?

Yes
No

How many years have you been in this role?

< 5yrs
≥ 5yrs
≥ 10yrs

Approximately how many hours per week do you provide breastfeeding support?

<10hrs
≥10hrs
>10hrs

What breastfeeding education have you attended over the past 3 years?

Australian Breastfeeding Association Courses
Advanced breastfeeding and lactation-Griffith University-online
Australian college of midwives ‘Supporting Women to Breastfeed’
Caper’s bookstores' events
Gold lactation online conferences
Health-e-learning lactation courses
Healthy Children’s Centre for Breastfeeding –online modules
iLactation online conferences
LCANZ-webinars
Master of midwifery (specialisation) Lactation Consultancy-Murdoch
Medscape
Praeclarus Press
The Royal College of Midwives –free midwifery/lactation education
WSLHD Breastfeeding workshop
Other
None

How many hours of breastfeeding education have you attended in the past year?

< 8hrs
≥ 8hrs
Other
None

Do you have IBCLC certification?

Yes- currently certified
Previously but not re-certified
Working towards
No

<u>Section C</u>
Discharge information

This section relates to the maternity notification system that you use to discharge mothers and babies. We are interested in how you use this system and what the definitions offered in this system mean to you.

Maternity notification system used at your workplace?

Cerner
e Maternity
Other free text:

How many maternal/infant discharges do you complete in an average week?

<10
≥10
>10

What time frame influences how you *report* 'infant feeding at discharge' when entering data into your electronic maternity database?

Most recent single-feeding episode
Most recent two or three feeding episodes
Feeding over the previous 12 hours
Feeding over the previous 24hrs
All feeding since birth
Other- free text

As a clinician what does 'full breastfeeding' at discharge mean to you?

Only breastfeeding and/or breast milk feeding on the day of discharge
Only breastfeeding and/or breast milk feeding over the last 12 hours
Only breastfeeding and/or breast milk feeding over the previous 24hrs
Only breastfeeding and/or breast milk feeding since birth
Other – free text

How well do you generally agree with the following statement:
'I often complete hospital maternity discharges without having had clinical contact with the woman or infant'

Agree, most of the time
Agree, some of the time
Neither agree nor disagree
Disagree, some of the time
Disagree, most of the time

If you have had no clinical contact with the mother and infant, where do you find 'feeding at discharge' information to discharge the mother and infant in your electronic data base?

e MR
e Maternity
Cerner
Mother
Staff
Postnatal charts
Other

How well do you generally agree with the following statement:
I feel confident the information that I enter for 'feeding at discharge' is accurate.

Agree, most of the time
Agree, some of the time
Neither agree nor disagree
Disagree, some of the time
Disagree, most of the time

<u>Section D</u>
Recommendations

The following question relates to your thoughts and recommendations as a clinician of a relevant definition of 'feeding at discharge'. Please keep in mind that some babies may be discharged days, weeks, or months after birth.

What <u>timeframe</u> do you think is most relevant to define 'feeding at discharge'?

Most recent single feeding episode
Most recent two or three feeding episodes
Feeding over the previous 12 hours
Feeding over the previous 24hrs
Feeding since birth

The following question relates to your thoughts and recommendations on the importance of understanding rates between hospitals of "exclusive breastfeeding" for women and babies in NSW. This is relevant for Baby Friendly Hospital Accreditation.

'Exclusive breastfeeding means that an infant receives only their mother's milk and necessary medications from birth'

How much do you agree that a separate question on exclusive breastfeeding at discharge would assist patient care?

Strongly disagree
disagree
neither agree/disagree
strongly agree
agree

Do have any further comments or suggestions on 'feeding at discharge'?

Free text
